# The Effects of Global Climate Warming on the Developmental Parameters of *Helicoverpa armigera* (Hübner, 1808) (Lepidoptera: Noctuidae)

**DOI:** 10.3390/insects15110888

**Published:** 2024-11-13

**Authors:** Zhiqian Liu, Biyu Liu, Huan Yu, Honghua Zhang, Zhipeng He, Zhihang Zhuo

**Affiliations:** College of Life Science, China West Normal University, Nanchong 637002, China; qnhtvxhp319123@foxmail.com (Z.L.); biyuliuql@foxmail.com (B.L.); 15082054112@163.com (H.Y.); honghua_zhang@foxmail.com (H.Z.); zhipeng_hh@foxmail.com (Z.H.)

**Keywords:** *Helicoverpa armigera* (Hübner, 1808), meta-analysis, climate change, integrated pest management, invasive insects

## Abstract

*Helicoverpa armigera* (Hübner, 1808) is a significant global agricultural pest, particularly posing a major threat during the boll-forming stage of cotton. In recent years, the severity of its damage has increased markedly, and its population dynamics and biological characteristics may be profoundly affected by global climate change. This study conducted a systematic meta-analysis to evaluate the life history traits of *H. armigera* under conditions of rising global temperatures, different photoperiods, and humidity levels. A comprehensive analysis of 26 related studies revealed that different developmental stages of *H. armigera* have distinct temperature requirements. When the temperature is within an optimal range (32 °C to 35 °C), the development rate of *H. armigera* accelerates, the life cycle shortens, and the reproductive capacity of female moths increases. However, when the temperature exceeds 35 °C, development slows, mortality rates increase, and the oviposition of female moths decreases significantly, indicating a negative impact of high temperatures on growth and reproduction. Overall, as the temperature rises above 20 °C, various physiological indicators of *H. armigera* significantly improve, and at 32 °C, the larval development period and overall life cycle reach their shortest duration. This meta-analysis provides new insights into the biological responses of *H. armigera* in the context of climate change and offers a scientific basis for future control strategies.

## Simple Summary

*Helicoverpa armigera* (Hübner, 1808), also known as the cotton bollworm, is a significant agricultural pest that impacts crops worldwide. This study explores the effects of global warming on the biological traits of this pest, with a particular focus on how temperature influences its development and reproduction. By analyzing data from 26 studies, we found that moderate temperature increases generally enhance the growth and reproductive rates of *H. armigera*. However, when temperatures exceed 35 °C, its survival and reproductive capacity begin to decline. Our findings indicate that, under climate change, moderate warming may boost the pest’s adaptability and amplify its threat to agriculture, whereas extreme heat could limit its spread. These insights are crucial for developing effective pest management strategies in a warming world.

## 1. Introduction

*Helicoverpa armigera*, commonly known as the cotton bollworm, is a significant agricultural pest belonging to the order Lepidoptera, and is widely distributed across warm and tropical regions globally [1]. It feeds on a variety of crops and wild plants, including cotton, maize, soybean, and vegetables, making it one of the most polyphagous pests [2,3]. The main damage caused by *H. armigera* occurs during its larval stage, where it feeds on plant tissues and fruits, severely impacting crop yield and quality [4,5]. Its broad host range and rapid reproductive capability lead to substantial economic losses and pose significant challenges in agricultural management [6]. In addition to direct crop damage, *H. armigera* is also noted for its resistance to insecticides [7,8,9]. Over the years, the agricultural sector has sought effective control strategies, including chemical control, biological control, and genetic modifications, to mitigate the damage caused by *H. armigera* and ensure stable and sustainable agricultural production [10,11].

Global climate change may significantly affect the geographical distribution of species, alter biodiversity, and impact interactions between species and ecosystems [12,13]. Due to their high reproductive capacity and population abundance, invasive species are often better equipped than native species to cope with climate change [14,15]. As temperatures rise, temperature-driven changes may alter ecosystem stability and sustain the multivoltine life cycles of insects [16]. Consequently, increasing temperatures may expand the global distribution of invasive crop pests, leading to more frequent outbreaks and increased damage to crops [17,18,19,20]. The establishment and spread of invasive pests in new environments are often attributed to the absence of natural enemies. Moreover, the lack of co-evolved host plants in invaded regions also plays an important role in facilitating pest invasions [21].

With the rapid changes in the Earth’s climate, the ability of invasive species to adjust their phenotypes is crucial for adapting to new environments [14]. Temperature is a key abiotic factor that regulates the development, reproductive capacity, and range of invasive species. The developmental rate of insects typically increases with rising temperatures [22]. High temperatures can accelerate the growth of *H. armigera* and shorten its life cycle, potentially leading to outbreaks [23,24,25,26]. Therefore, it is essential to assess the impact of temperature changes on the growth and development of *H. armigera*.

Although numerous studies have reported the effects of temperature on *H. armigera*, no systematic quantitative analysis has been conducted to comprehensively evaluate these effects. In this study, we use a meta-analysis to synthesize and evaluate the physiological responses of *H. armigera* under different temperature conditions, as well as its stress responses at various developmental stages to changes in temperature.

## 2. Materials and Methods

### 2.1. Literature Search

To systematically assess the impact of global climate change on the biological traits of *H. armigera*, this study employed a systematic literature search approach, collecting relevant studies from multiple databases. The literature search was conducted between August and October 2024, with the primary databases being Web of Science, PubMed, Scopus, and CNKI. Additionally, we manually reviewed the reference lists of relevant review articles to supplement studies not indexed in the databases. The search keywords included “*Helicoverpa armigera*”, “climate change” (or “global warming”), “temperature”, “humidity” (or “precipitation”), and “biological traits” (or “life history traits”, “development”, “reproduction”, etc.). Keywords were combined using Boolean operators (AND, OR) to ensure comprehensive coverage of the relevant literature [27].

The literature screening process was carried out in two steps: first, an initial screening based on titles and abstracts was conducted to exclude studies unrelated to climate change or *H. armigera*; second, full-text reviews of the remaining studies were performed to further eliminate those that did not meet the inclusion criteria. The inclusion criteria for studies were as follows: The study explicitly assessed the effects of climate factors, such as temperature and relative humidity, on the biological traits of *H. armigera* (e.g., development rate, reproductive capacity, and survival rate). Experimental designs included a control group and clearly reported specific experimental conditions (e.g., temperature range, humidity conditions). Studies provided data suitable for meta-analysis (e.g., means, standard deviations, and sample sizes) or data that could be extracted from figures and tables. If only the standard error (SE) was provided, it was converted to the standard deviation (SD) using the formula of dividing the standard error by the square root of the sample size [28].

Exclusion criteria included: studies without a control experiment or those limited to theoretical model simulations; studies that did not address the biological traits of *H. armigera* or climate factors; and studies lacking sufficient statistical data for meta-analysis.

### 2.2. Inclusion Criteria

Data were extracted from studies that met the inclusion criteria. The key variables extracted included: temperature (different experimental temperature levels in °C); relative humidity; and biological traits (such as development rate, generation time, and reproductive capacity, e.g., oviposition rate). When necessary statistical information, such as standard deviation or sample size, was not directly provided, we used graphical data extraction tools (e.g., WebPlotDigitizer) to extract data from figures and charts. For experiments with multiple variables or multiple treatment groups, different treatment outcomes were considered as independent effect sizes.

### 2.3. Statistical Analysis

In this study, we used the “rma.mv” function from the R package “metafor” (version 4.3) to perform the following steps [29]. First, we calculated the relative risk (RR^+^) using a random-effects model and estimated variance between studies (I^2^) using the restricted maximum likelihood (REML) method [30]. Based on the I^2^ value, we introduced moderator variables. Next, we computed the overall mean effect size of all temperature treatment groups using a random-effects model. Finally, we conducted all statistical tests, including the analysis of average effect size, 95% confidence intervals (CI), Qt, and I^2^ [31]. Meta-analyses were performed separately to determine the extent to which different independent variables were affected by temperature, thereby assessing the impact of temperature on different developmental stages of *H. armigera*.

This study employed both random-effects and fixed-effects models to integrate results from different studies. Due to substantial heterogeneity among studies (e.g., differences in experimental locations and treatment approaches), the random-effects model was more suitable for explaining variability between studies. During the analysis, climate-related factors (temperature, relative humidity) were treated as independent variables, while the biological traits of *H. armigera* were treated as dependent variables. Effect sizes were measured using the standardized mean difference (Log Response Ratio, LRR) [32], which indicates the difference between climate factor treatment groups and control groups regarding the biological traits of *H. armigera*, with statistical significance tested through 95% confidence intervals.

To evaluate the impact of different climate factors on the biological traits of *H. armigera*, subgroup analyses were conducted for temperature and relative humidity. Heterogeneity among studies was assessed using the Q statistic and I^2^ index. The I^2^ value reflects the extent of heterogeneity; a high I^2^ value indicates significant differences among studies. The heterogeneity statistic tests the weighted sum of squares for a k-1 distribution. When the 95% confidence interval of the effect size includes 0, it indicates that the effect size of the experimental group is equal to that of the reference group (*p* > 0.05). When the 95% confidence interval is entirely >0, the experimental group effect size is greater than the control group (*p* < 0.05). Conversely, when the 95% confidence interval is entirely <0, the experimental group effect size is smaller than the control group (*p* < 0.05). Based on the cumulative effect size and its significance relative to zero, as well as the *p*-value for Qt, we determined whether moderator variables should be included. Potential moderators considered included the effects of humidity and photoperiod on cumulative effect size [33]. Additionally, temperature data were treated as continuous variables to determine their impact on mean effect size.

In meta-analysis, overall heterogeneity is divided into variance explained by categorical factors (between-group heterogeneity) and residual variance (within-group heterogeneity), with significance determined through a k-1 test. Furthermore, we evaluated potential publication bias through funnel plots and radial plots [34]. If significant bias was detected, adjustments were made using the “trim and fill” method.

## 3. Results

### 3.1. Literature Search and Screening Results

Through a systematic search, we identified a total of 428 potentially relevant studies. During the initial screening process, 153 studies were excluded as they were not relevant to the research topic. After conducting full-text reviews of the remaining 375 studies, 26 studies met the inclusion criteria. These studies covered the effects of different climate factors (temperature, humidity) on the biological traits of *H. armigera*. From the 26 studies that met the inclusion criteria, we collected a total of 525 datasets, which included the following developmental stages: first instar (n = 26), second instar (n = 26), third instar (n = 26), fourth instar (n = 26), fifth instar (n = 26), adult longevity (female) (n = 35), adult longevity (male) (n = 35), egg stage (n = 38), fecundity (n = 20), life cycle (n = 40), oviposition period (n = 35), pre-oviposition period (n = 33), pupal stage (n = 50), larval stage (n = 109), and the overall impact of temperature changes on *H. armigera* (Table 1).

### 3.2. Overall Impact of Temperature on Biological Traits

**Figure 1:**
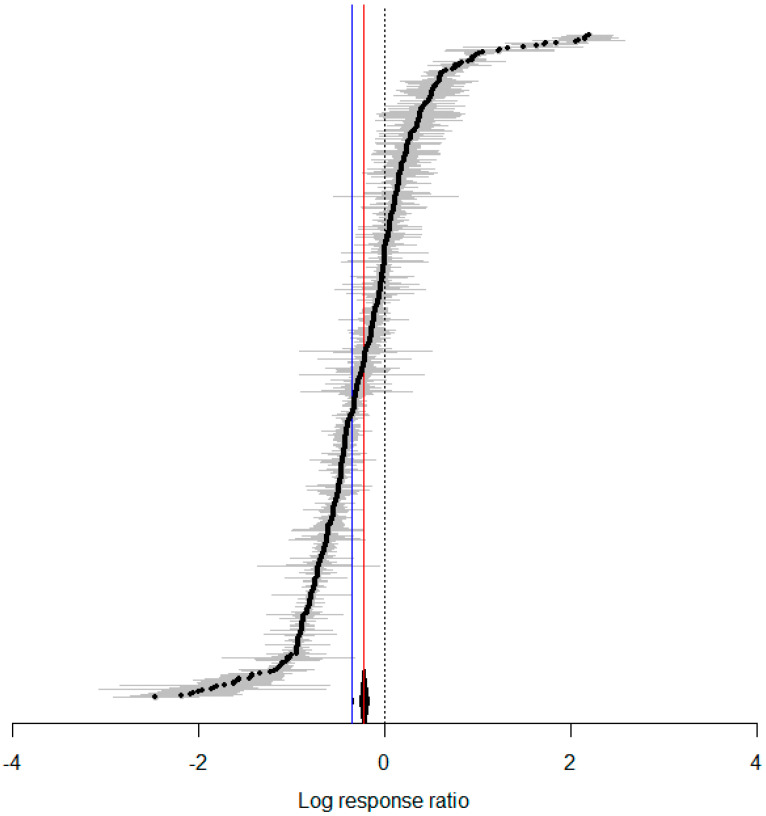
Forest plot of the effects of temperature variation on *H. armigera*. (The red line represents the calculated results of the random-effects model, with a cumulative effect size of −0.2231 and 95% confidence interval ranging from −0.2747 to −0.1716. The solid blue line represents the calculated results of the fixed-effects model, with a cumulative effect size of −0.3488 and 95% confidence interval ranging from −0.3513 to −0.3463.)

**Figure 2:**
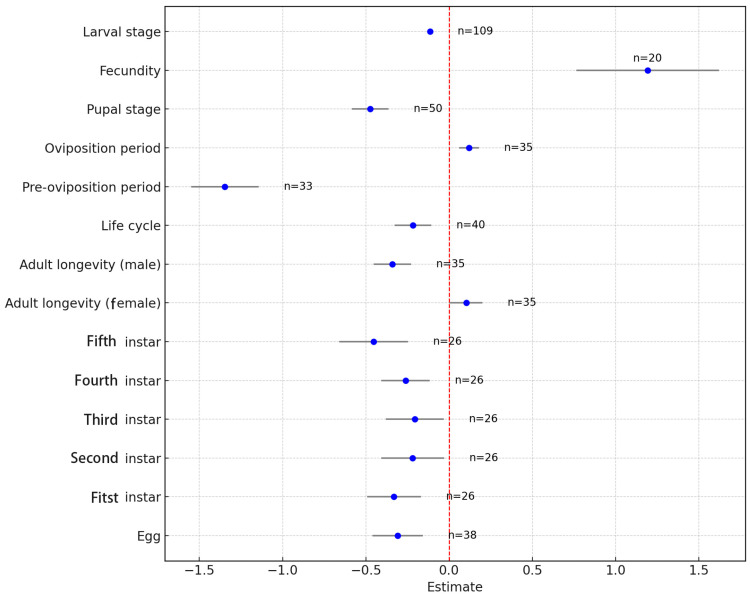
Forest plot of the effects of temperature variation on various physiological indicators of *H. armigera*.

**Figure 3:**
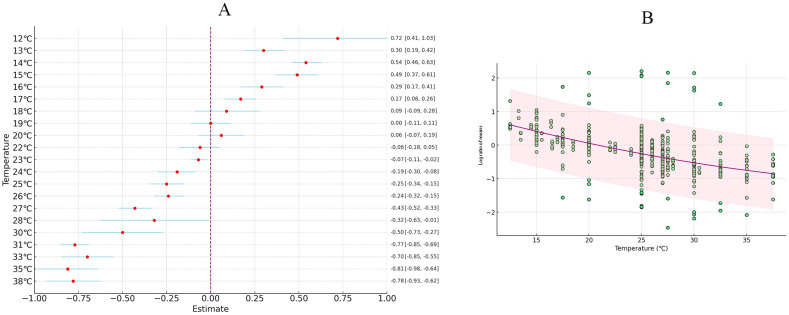
The impact of temperature variation on *H. armigera*. (Panel (**A**) depicts the changes in physiological properties of *H. armigera* with increasing temperature; Panel (**B**) shows the temperature range curve for the optimal growth of *H. armigera.*).

A meta-analysis was conducted by synthesizing data from various studies, and the results showed that increased temperature generally enhanced the fitness of *H. armigera*. The overall mean effect size was −0.2231 (CI: −0.2747 to −0.1716; Figure 1, Table 2). The oviposition period lengthened with increasing temperature, female adult longevity increased, and female oviposition also increased as temperature rose, while other dependent variables related to *H. armigera* showed significant decreases with rising temperatures (Figure 2). When temperature was treated as a continuous variable, it was observed that physiological indicators of *H. armigera* significantly improved as temperature rose beyond 20 °C (Figure 3A). The physiological activity peaked when temperatures reached 32–35 °C (Figure 3B).

### 3.3. Effect of Temperature on Developmental Duration

**Figure 4:**
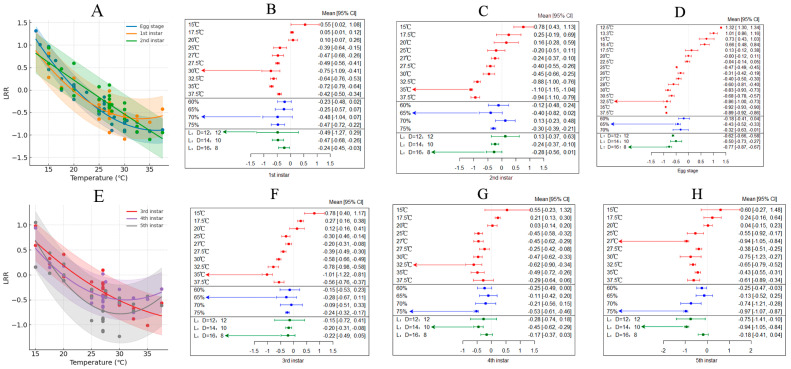
The impact of temperature on various developmental stages of *H. armigera*. ((**A**) illustrates the changes in developmental time of egg stage to second instar larvae with temperature variations; (**B**–**D**) depict the responses of egg stage to second instar larvae to external environmental conditions; (**E**) illustrates the changes in developmental time of third to fifth instar larvae with temperature variations; (**F**–**H**) depict the responses of third to fifth instar larvae to external environmental conditions.)

Various studies have shown that increased temperatures lead to a reduction in the developmental time of *H. armigera* eggs, with an overall average effect size of −0.3091 (95% CI: −0.4612; −0.1571; Figure S1, Table 2). Within the temperature range of 12–38 °C, the developmental time of first instar larvae significantly decreased as temperatures rose (Figure 4A). Results from both the random-effects model and the fixed-effects model indicated that *Q* (df = 48) = 129,125.6231, *p* < 0.0001, suggesting that inter-group variability affects the cumulative effect size, thereby necessitating the inclusion of explanatory variables such as humidity and photoperiod (Figure 4B). The optimal temperature for egg development was identified as 32.5 °C. Additionally, the impacts of varying relative humidity levels and photoperiods on egg development differ, with a relative humidity of 65% and a photoperiod of L:D = 16:8 being most favorable for egg development.

Various studies have shown that increased temperatures lead to a shortened developmental period for first instar *H. armigera* larvae, with an overall average effect size of -0.3091 (95% CI: −0.4928; −0.169; Figure S2, Table 2). Within the temperature range of 15–38 °C, the developmental time of first instar larvae significantly decreased as temperatures rose (Figure 4A). The results from both the random-effects model and the fixed-effects model indicated that *Q* (df = 25) = 1916.5405, *p* < 0.0001, suggesting the presence of inter-group differences affecting the cumulative effect size, which necessitates the introduction of explanatory variables such as humidity and photoperiod (Figure 4C). The optimal temperature for egg development was 30 °C. The effects of different relative humidity levels and photoperiods on egg development were significant; specifically, a relative humidity of 70% and a photoperiod of L:D = 12:12 were optimal for first instar development.

In the studies examined, elevated temperatures resulted in a shortened developmental period for second instar *H. armigera* larvae, with an overall average effect size of −0.2187 (95% CI: −0.4079; −0.0296; Figure S3, Table 2). Within the temperature threshold of 15–38 °C, an increase in temperature significantly reduced the developmental time of first instar larvae (Figure 4A). Calculations from both the random-effects model and the fixed-effects model revealed that *Q* (df = 25) = 5202.7682, *p* < 0.0001, indicating inter-group variability that affects the cumulative effect size, thereby necessitating the incorporation of explanatory variables such as humidity and photoperiod (Figure 4D). The optimal temperature for egg development was identified as 35 °C. Furthermore, the effects of varying relative humidity levels and photoperiods on egg development were notable, with a relative humidity of 65% and a photoperiod of L:D = 16:8 being most conducive to the development of second instar larvae.

In the studies analyzed, increased temperatures resulted in a shortened developmental period for third instar *H. armigera* larvae, with an overall average effect size of −0.2058 (95% CI: −0.3792; −0.0324; Figure S4, Table 2). Within the temperature range of 15–38 °C, the developmental time of third instar larvae significantly decreased as temperatures rose (Figure 4E). Results from both the random-effects model and the fixed-effects model indicated that *Q* (df = 48) = 1119.8463, *p* < 0.0001, suggesting inter-group variability affects the cumulative effect size, necessitating the inclusion of explanatory variables such as humidity and photoperiod (Figure 4F). The optimal temperature for the development of third instar larvae was identified as 35 °C. Additionally, the impacts of varying relative humidity levels and photoperiods on egg development differ, with a relative humidity of 65% and a photoperiod of L:D = 16:8 being most favorable for the development of third instar larvae.

Various studies have shown that increased temperatures lead to a reduction in the developmental time of fourth instar *H. armigera* larvae, with an overall average effect size of −0.2614 (95% CI: −0.4065; −0.1163; Figure S5, Table 2). Within the temperature range of 15–38 °C, the developmental time of third instar larvae significantly decreased as temperatures rose (Figure 4E). Results from both the random-effects model and the fixed-effects model indicated that *Q* (df = 25) = 1274.5347, *p* < 0.0001, suggesting that inter-group variability affects the cumulative effect size, necessitating the inclusion of explanatory variables such as humidity and photoperiod (see Figure 4G). The optimal temperature for the development of fourth instar larvae was identified as 32 °C. Additionally, the impacts of varying relative humidity levels and photoperiods on egg development differ, with a relative humidity of 75% and a photoperiod of L:D = 14:10 being most favorable for the development of fourth instar larvae.

Various studies have shown that increased temperatures lead to a reduction in the developmental time of fifth instar *H. armigera* larvae, with an overall average effect size of −0.4531 (95% CI: −0.6587; −0.2475; Figure S6, Table 2). Within the temperature range of 15–378 °C, the developmental time of third instar larvae significantly decreased as temperatures rose (Figure 4E). Results from both the random-effects model and the fixed-effects model indicated that *Q* (df = 25) = 1420.3349, *p* < 0.0001, suggesting that inter-group variability affects the cumulative effect size, necessitating the inclusion of explanatory variables such as humidity and photoperiod (Figure 4H). The optimal temperature for the development of fifth instar larvae was identified as 27 °C. Additionally, the impacts of varying relative humidity levels and photoperiods on egg development differ, with a relative humidity of 75% and a photoperiod of L:D = 14:10 being most favorable for the development of fifth instar larvae.

### 3.4. The Impact of Temperature Variation on Oviposition Physiology of H. armigera

**Figure 5:**
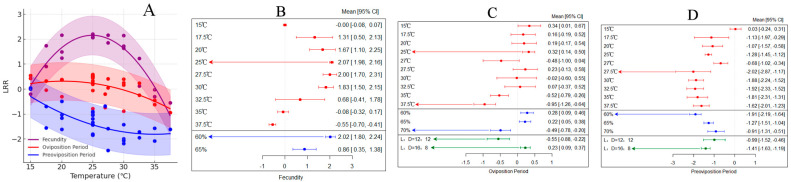
The impact of temperature on the oviposition physiology of *H*. *armigera*. ((**A**) illustrates the variation in oviposition physiology of female *H*. *armigera* with temperature changes; (**B**) depicts the response of oviposition quantity of female *H*. *armigera* to changes in external environmental conditions; (**C**) shows the response of oviposition period of female *H*. *armigera* to changes in external environmental conditions; (**D**) demonstrates the response of pre-oviposition period of female *H*. *armigera* to changes in external environmental conditions.)

Various studies have shown that increased temperatures lead to a rise in the egg-laying capacity of female *H. armigera*, with an overall average effect size of 1.1927 (95% CI: 0.7645; 1.6209; Figure S7, Table 2). Within the temperature range of 15–38 °C, as temperatures rose, the reproductive capacity of *H. armigera* significantly increased (Figure 5A). Results from both the random-effects model and the fixed-effects model indicate that *Q* (df = 19) = 2035.8560, *p* < 0.0001, suggesting that inter-group heterogeneity affects the cumulative effect size, thus necessitating the inclusion of explanatory variables (Figure 5B). The highest egg production by female *H. armigera* occured at a temperature of 25 °C and a relative humidity of 60%.

In various studies, increased temperature led to an extension of the oviposition period in female *H. armigera*, with an average effect size of 0.1203 (CI: 0.0606; 0.1799; Figure S8, Table 2). Within the temperature range of 15–38 °C, the oviposition period of *H. armigera* significantly increased with rising temperatures (Figure 5A). The results from both random-effects and fixed-effects models indicated that *Q* (df = 34) = 226.5584, *p* < 0.0001, suggesting the presence of inter-group heterogeneity affecting the cumulative effect size, thus requiring the inclusion of relevant explanatory variables (Figure 5C). The optimal oviposition period for female *H. armigera* occured at a temperature of 25 °C, with a relative humidity of 75% and L:D = 16:8.

In various studies, increased temperature significantly shortened the pre-oviposition of female *H. armigera*, with an average effect size of −1.3465 (CI: −1.5491; −1.1438; Figure S9, Table 2). Within the temperature range of 15–38 °C, the pre-oviposition of *H. armigera* significantly decreased with rising temperatures (Figure 5A). The results from both random-effects and fixed-effects models indicated that *Q* (df = 32) = 250.5705, *p* < 0.0001, suggesting the presence of inter-group heterogeneity that affects the cumulative effect size, thus requiring the inclusion of relevant explanatory variables (Figure 5D). Different photoperiods and humidity levels have varying impacts on the pre-oviposition of *H. armigera*. When the temperature reached 27 °C, with a relative humidity of 60% and L:D = 16:8, the conditions for the pre-oviposition of *H. armigera* were most suitable.

### 3.5. Effect of Temperature on the Lifespan of Female and Male Adults

**Figure 6:**
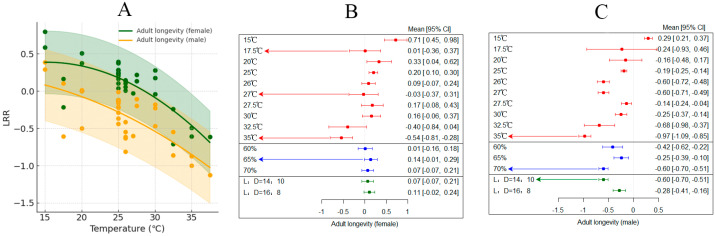
The impact of temperature on the lifespan of female and male adults of *H*. *armigera*. ((**A**) explains the changes in the lifespan of male and female adults with temperature changes; (**B**) depicts the response of adult female *H*. *armigera* longevity to changes in external environmental conditions; (**C**) shows the response of adult male *H*. *armigera* longevity to changes in external environmental conditions.)

In various studies, increased temperature significantly extended the lifespan of adult female *H. armigera*, with an average effect size of 0.104 (CI: 0.001; 0.2070; Figure S10 Table 2). Within the temperature range of 15–35 °C, the lifespan of adult females significantly increased as the temperature rose (Figure 6A). The results from both random-effects and fixed-effects models indicated that *Q* (df = 34) = 92.4024, *p* < 0.0001, suggesting the presence of inter-group heterogeneity that affects the cumulative effect size, thus necessitating the inclusion of relevant explanatory variables (Figure 6B). Different photoperiods and humidity levels have varying impacts on the lifespan of adult females of *H. armigera*. When the temperature reached 35 °C and relative humidity was 65%, the lifespan of adult females was the shortest.

In various studies, increased temperature led to a decrease in the lifespan of adult male *H. armigera*, with an average effect size of −0.3409 (CI: −0.4535; −0.2283; Figure S11, Table 2). Within the temperature range of 15–35 °C, the lifespan of adult males significantly decreased as the temperature rose (Figure 6A). The results from both random-effects and fixed-effects models indicated that *Q* (df = 34) = 881.2395, *p* < 0.0001, suggesting the presence of inter-group heterogeneity that affects the cumulative effect size, thus necessitating the inclusion of relevant explanatory variables (Figure 6C). Different photoperiods and humidity levels have varying impacts on the lifespan of adult males of *H. armigera*. When the temperature reached 35 °C, with a relative humidity of 70% and L:D = 14:10, the lifespan of adult males was the shortest.

### 3.6. The Effects of Temperature on the Larval Stage, Life Cycle, and Pupal Stage

**Figure 7:**
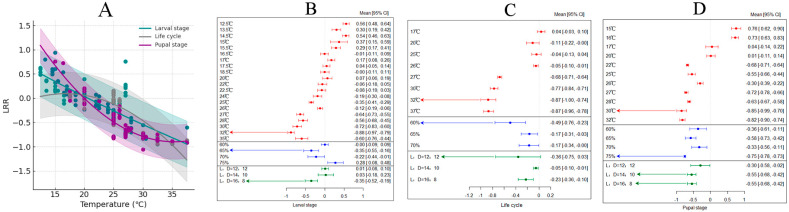
The impact of temperature variations on the larval development, complete life cycle, and pupal stage of *H*. *armigera*. ((**A**) describes the changes in the larval stage, life cycle, and pupal stage of *H*. *armigera* with temperature changes; (**B**) depicts the response of the larval stage of *H*. *armigera* to changes in external environmental conditions; (**C**) shows the response of the life cycle of *H*. *armigera* to changes in external environmental conditions; (**D**) demonstrates the response of the pupal stage of *H*. *armigera* to changes in external environmental conditions.)

Research shows that increased temperature leads to a reduction in the developmental duration of the entire larval stage of *H. armigera*, with an overall average effect size of −0.1144 (CI: −0.1291; −0.0996; Figure S12, Table 2). Within the temperature threshold range of 12–35 °C, the developmental time of the larval stage of *H. armigera* significantly decreases with rising temperatures (Figure 7A). The results from both the random-effects model and fixed-effects model indicate that *Q* (df = 108) = 3395.4298, *p* < 0.0001, suggesting significant inter-group heterogeneity that affects the cumulative effect size, necessitating the inclusion of explanatory variables (Figure 7B). Different photoperiods and humidity conditions have varying effects on the larval stage of *H. armigera*. When the temperature reaches 32 °C, with a relative humidity of 65% and L:D = 16:8, the developmental duration of the entire larval stage of *H. armigera* is shortest.

Research indicates that increased temperature accelerates the generation cycle frequency of *H. armigera*, with an overall average effect size of −0.2171 (CI −0.3265; −0.1077; Figure S13, Table 2). Within the temperature threshold range of 17–37 °C, the developmental time of the larval stage of *H. armigera* significantly decreases with rising temperatures (Figure 7A). The results from both the random-effects model and fixed-effects model show that *Q* (df = 39) = 6001.3758, *p* < 0.0001, indicating that inter-group heterogeneity significantly affects the cumulative effect size, thus necessitating the inclusion of explanatory variables (Figure 7C). Different photoperiods and humidity conditions have varying effects on the generation cycle of *H. armigera*. When the temperature reaches 32 °C, with a relative humidity of 60% and L:D = 12:12, the time required for the entire life cycle of *H. armigera* is shortest.

Research indicates that increased temperature leads to a reduction in the pupal period of *H. armigera*, with an overall average effect size of −0.4748 (CI: −0.5849; −0.3648; Figure S14, Table 2). Within the temperature threshold range of 15–32 °C, the duration of the pupal stage significantly decreases as the temperature rises (Figure 7A). The results from both the random-effects model and fixed-effects model indicate that *Q* (df = 38) = 5646.9218, *p* < 0.0001, suggesting that inter-group differences significantly affect the cumulative effect size, thus necessitating the inclusion of explanatory variables (Figure 7D). The findings reveal that different humidity and photoperiod conditions also have varying impacts on the pupal period of *H. armigera.* When the temperature reaches 30 °C, with a relative humidity of 75% and L:D = 16:8, the conditions are most favorable for the development of the pupal stage of *H. armigera*.

### 3.7. Model Testing

**Figure 8:**
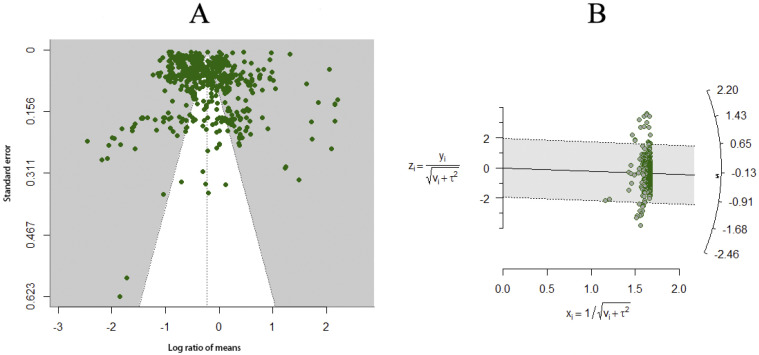
(**A**) is a funnel chart; (**B**) is a radar chart.

We utilized funnel plots and radar charts to assess whether the results were influenced by publication bias, and we calculated the fail-safe N to verify the reliability of our findings. The results indicated that the funnel plot (Figure 8A) (*z* = 1.0324, *p* = 0.3019), the radar chart (Figure 8B), and the fail-safe N (N = 75123) all demonstrate that our results are reliable.

## 4. Discussion

The meta-analysis in this study was conducted through systematic literature searches and screening, ultimately including 26 studies that examined the effects of different temperature scenarios on the biological traits of *H. armigera*. The literature screening results showed that research on the impact of temperature on the biological traits of *H. armigera* was concentrated in regions such as East Asia, Southeast Asia, South Asia, and West Africa, which are the major occurrence areas of *H. armigera* [35,36]. The studies involved mostly laboratory-controlled trials, with a few field experiments. The screening results indicated that although temperature is a widely influential environmental factor on *H. armigera*, experimental conditions, species, and reporting methods varied among the studies, leading to a certain degree of heterogeneity. To address this heterogeneity, we used a random-effects model for analysis and further explored the effects of different temperatures on various biological traits of *H. armigera* through subgroup analysis.

Temperature significantly regulated the biological traits of *H. armigera*. According to the meta-analysis results, higher temperatures accelerated the development rate and reproductive capacity of *H. armigera*, but this positive effect weakened or even reversed under extreme high temperatures. This finding is consistent with previous studies [37,38], indicating that *H. armigera* can shorten its life cycle and increase population numbers within a suitable temperature range. It is noteworthy that when the temperature exceeds the upper tolerance limit (above 35 °C), both the developmental rate and survival rate of *H. armigera* declined. This is related to the negative effects of temperature stress on the physiological processes of insects [39,40], suggesting that extreme high temperatures under climate change may inhibit the expansion of *H. armigera*.

The response of different larval stages to temperature changes was not uniform. According to the meta-analysis results, the developmental rate of all larval stages increased with temperature, but their sensitivity to temperature varied. The larval stage was most sensitive to temperature changes, with a significantly accelerated development rate at higher temperatures; however, larval mortality also increased under high temperatures. This could be due to the increased survival pressure faced by *H. armigera* during the larval stage, as they need to adapt to rapid development and extreme environmental changes. In the later larval stages, the impact of temperature was less pronounced, indicating that *H. armigera* gradually developed greater temperature tolerance as they matured, showing resilience to environmental changes.

Temperature also had a significant impact on the oviposition physiology of female *H. armigera*. The meta-analysis showed that rising temperatures significantly increased the oviposition rate of female adults, particularly within the optimal temperature range of 25 °C to 27 °C, where oviposition reached its peak. However, when temperatures rose above 32 °C, the oviposition rate gradually decreased, indicating that high temperatures had an inhibitory effect on the reproductive capacity of *H. armigera*. High temperatures negatively affected the physiological activities of female adults, such as delayed ovarian development or reduced egg quality, consistent with the hypothesis that high-temperature stress impairs the reproductive system of insects [41]. Therefore, in the context of climate warming, moderate temperature increases may promote population growth, but extreme high temperatures may inhibit population reproduction.

The effect of temperature on the developmental period of *H. armigera* exhibited significant stage-specific characteristics [42]. The developmental period of *H. armigera* shortened as temperature increased, indicating that temperature accelerated its development process; however, this effect showed a certain delay in the adult stage. According to the meta-analysis results, the temperature response of *H. armigera* during different developmental stages showed cumulative effects—temperature increases accelerated early larval development but had negative effects on adult survival and behavior, particularly under high-temperature conditions where adult longevity significantly decreased. This is closely related to the inhibitory effects of high temperatures on energy metabolism and behavioral capacity in insects [43,44]. Therefore, temperature had a positive regulatory effect on the developmental period of *H. armigera*, but adult survival in high-temperature conditions was reduced, potentially impacting its long-distance migration and dispersal capacity.

Insects are known to be ectothermic animals, and changes in insect population distribution are strongly influenced by temperature [45,46]. As a result, the distribution patterns of many insect species are significantly affected by global warming, leading to expanded ranges and shifts [47,48,49]. Under such conditions, temperature may be a major driver of the survival, development, and reproduction of *H. armigera*. Temperature has a significant impact on the growth and development cycle of *H. armigera*, promoting growth within a suitable temperature range. We also analyzed the relationship between different developmental stages of *H. armigera* and temperature, obtaining corresponding response curves. It was observed that all developmental stages of *H. armigera* varied with temperature changes (Table 3). Continued global warming will greatly alter ecosystem function and structure, leading to changes in habitat distribution [50,51]. The increased incidence and damage of insect pests and the expansion of their distribution will have profound impacts on agricultural production. This study emphasizes that under future warming conditions, the adaptability of *H. armigera* is expected to increase gradually. This research provides valuable information for policymakers to develop appropriate pest management strategies to prevent widespread economic losses that could result from climate warming.

## 5. Conclusions

This study systematically assessed the impact of temperature changes on the biological traits of *H. armigera* under global climate change through meta-analysis. The results showed that temperature increases within a suitable range (32 °C to 35 °C) significantly accelerated the development rate, shortened the life cycle, and increased the oviposition rate of female adults. However, under extreme high-temperature conditions (above 35 °C), the developmental rate of *H. armigera* slowed, mortality increased, and the reproductive capacity of female adults was significantly inhibited, indicating an upper limit to the temperature’s effect on *H. armigera*. The sensitivity of different developmental stages of *H. armigera* to temperature changes varied; larvae were most sensitive to high temperatures, while adult survival was lower under high temperatures, potentially impacting the expansion and migration capacity of the population. These findings suggest that temperature changes can have significant effects on the development and distribution of *H. armigera*, as was shown for many other insects, indicating that similar temperature-dependent trends may also be observed in other species with similar taxonomic characteristics. Under moderate temperature increases, developmental acceleration and increased reproductive rates are likely to occur, whereas extreme high temperatures could hinder growth and reduce population viability. This insight provides a broader understanding of how temperature may influence the biological processes and geographical distribution of similar insect species under changing climate conditions.

## Figures and Tables

**Table 1:** Dataset summary based on inclusion criteria.

Temperature Range	Control Temperature	Number of Datasets (n)	Variable
15–38 °C	20	26	First instar
15–38 °C	20	26	Second instar
15–38 °C	20	26	Third instar
15–38 °C	20	26	Fourth instar
15–38 °C	20	26	Fifth instar
15–35 °C	20	35	Adult longevity (female)
15–35 °C	20	35	Adult longevity (male)
12–38 °C	20	38	Egg stage
15–38 °C	20	20	Fecundity
17–38 °C	20	40	Life cycle
15–38 °C	20	35	Oviposition period
15–38 °C	20	33	Pre-oviposition period
15–32 °C	20	50	Pupal stage
12–35 °C	20	109	Larval stage
12–38 °C	20	525	*H. armigera*

**Table 2:** Random-effects model calculation results.

Variable	Estimate	se	z	*p*	CI.lb	CI.Ub	LogLik	AIC	BIC
*Helicoverpa armigera (Hübner)*	−0.2231	0.0263	−8.4808	<0.0001	−0.2747	−0.1716	−503.4509	1010.9017	1019.4662
Egg	−0.3091	0.0776	−3.9845	<0.0001	−0.4612	−0.1571	−38.7345	81.469	85.2114
First instar	−0.3309	0.0826	−4.0056	<0.0001	−0.4928	−0.169	−13.8564	31.7128	34.1505
Second instar	−0.2187	0.0965	−2.2668	<0.0001	−0.4079	−0.0296	−14.7668	39.5337	41.9714
Third instar	−0.2058	0.0885	−2.3257	<0.0001	−0.3792	−0.0324	−15.5972	35.1943	37.6321
Fourth instar	−0.2614	0.074	−3.5314	<0.0001	−0.4065	−0.1163	−11.0511	26.1022	28.5399
Fifth instar	−0.4531	0.1049	−4.3194	<0.0001	−0.6587	−0.2475	−19.8645	43.729	46.1668
Adult longevity (female)	0.104	0.0525	1.979	<0.0478	0.001	0.207	−9.2596	22.5192	22.9063
Adult longevity (male)	−0.3409	0.0574	−5.9345	<0.0001	−0.4535	−0.2283	−11.8951	27.7901	30.8429
Life cycle	−0.2171	0.0558	−3.8902	0.0001	−0.3265	−0.1077	−14.7148	33.4296	36.7567
Pre-oviposition period	−1.3465	0.1034	−13.024	<0.0001	−1.5491	−1.1438	−28.7279	61.4558	64.3873
Oviposition period	0.1203	0.0304	3.9516	<0.0001	0.0606	0.1799	−86.4086	174.8172	176.3725
Pupal stage	−0.4748	0.0561	−8.4578	<0.0001	−0.5849	−0.3648	−24.4508	52.9017	56.6853
Fecundity	1.1927	0.2185	5.4593	<0.0001	0.7645	1.6209	−26.4847	56.9694	58.8583
Larval stage	−0.1144	0.0075	−15.2245	<0.0001	−0.1291	−0.0996	−1526.7594	3055.4989	3058.1902

**Table 3:** The optimal external environment for each developmental stage of *H. armigera*.

Growth History	Optimal Survival Temperature	Optimum RH for Survival	Optimum Photoperiod for Survival
Egg	32 °C	65%	L:D = 16:8
First instar	30 °C	70%	L:D = 12:12
Second instar	35 °C	65%	L:D = 16:8
Third instar	35 °C	65%	L:D = 16:8
Fourth instar	32 °C	75%	L:D = 14:10
Fifth instar	27 °C	75%	L:D = 14:10
Adult longevity (female)	35 °C	65%	L:D = 14:10
Adult longevity (male)	35 °C	70%	L:D = 14:10
Life cycle	32 °C	60%	L:D = 12:12
Pre-oviposition period	27 °C	60%	L:D = 16:8
Oviposition period	25 °C	75%	L:D = 16:8
Pupal stage	30 °C	75%	L:D = 12:12
Fecundity	25 °C	60%	-
Larval stage	32 °C	65%	L:D = 16:8

## Data Availability

The data supporting the results are available in a public repository at: https://doi.org/10.6084/m9.figshare.27684738, accessed on 11 October 2024.

## Supplementary Materials

The following supporting information can be downloaded at: https://www.mdpi.com/article/10.3390/insects15110888/s1, Figure S1: Comparison of effect sizes between random-effects and fixed-effects models; Figure S2: Comparison of effect sizes between random effects and fixed-effects models; Figure S3: Comparison of effect sizes between random-effects and fixed-effects models; Figure S4: Comparison of effect sizes between random-effects and fixed-effects models; Figure S5: Comparison of effect sizes between random-effects and fixed-effects models; Figure S6: Comparison of effect sizes between random-effects and fixed-effects models; Figure S7: Comparison of effect sizes between random-effects and fixed-effects models; Figure S8: Comparison of effect sizes between random-effects and fixed-effects models; Figure S9: Comparison of effect sizes between random-effects and fixed-effects models; Figure S10: Comparison of effect sizes between random-effects and fixed-effects models; Figure S11: Comparison of effect sizes between randomeffects and fixed-effects models; Figure S12: Comparison of effect sizes between random-effects and fixed-effects models; Figure S13: Comparison of effect sizes between random-effects and fixedeffects models; Figure S14: Comparison of effect sizes between random-effects and fixed-effects models.
