# Supplementary material for: The Effects of Global Climate Warming on the Developmental Parameters of Helicoverpa armigera (Hübner, 1808) (Lepidoptera: Noctuidae)
Source: Insects. 2024 Nov 13;15(11):888. doi: 10.3390/insects15110888 (PMC11594439; doi:10.3390/insects15110888)
Supplement: Supplementary file 1 [file insects-15-00888-s001.zip › insects-3304713-supplementary.pdf]

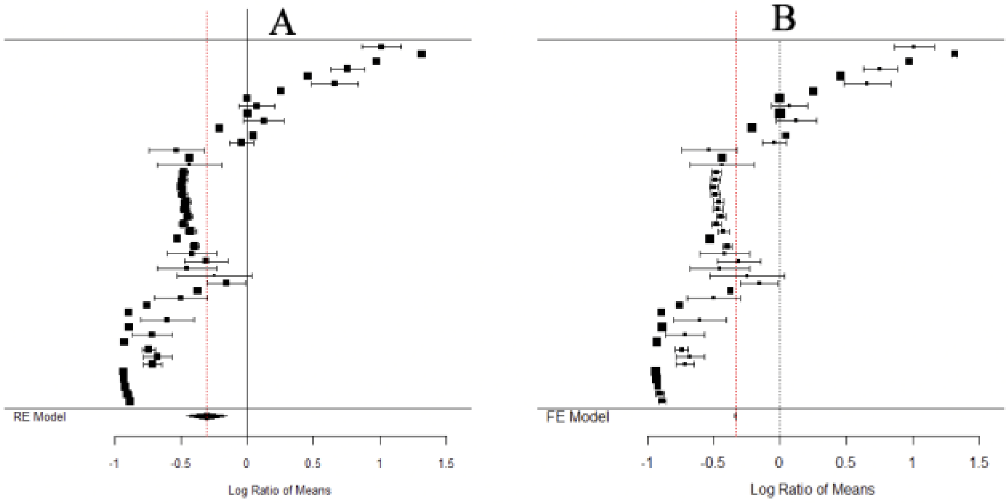

Fig. S1 Comparison of effect sizes between random-effects and fixed-effects models (Panel A displays the results of the random-effects model calculation, E = -0.3091, 95% confidence interval ranging from -0.4612 to -0.1571, Q (df = 48) = 129125.6231, $p <$ 0.0001, I^2 = 99.96%. Panel B shows the results of the fixed-effects model calculation, E = -0.3349, 95% confidence interval ranging from -0.3381 to -0.3318, Q (df = 48) = 129125.6231, $p < 0.0001$, I^2 = 99.96%. Black squares represent the values of the cumulative effect size for each variable. Black horizontal lines represent the 95% confidence interval. Red solid lines indicate the cumulative effect values.)

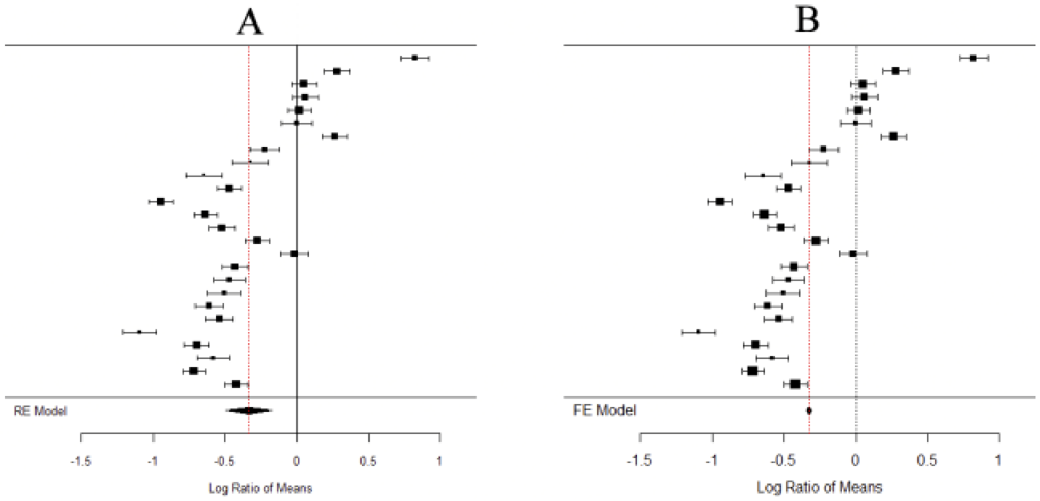

Fig. S2 Comparison of effect sizes between random-effects and fixed-effects models (Panel A displays the results of the random-effects model calculation, E = -0.3309, 95% confidence interval ranging from -0.4928 to -0.1690, Q (df = 25) = 1916.5405, $p <$ 0.0001, $I^2 = 98.72\%$. Panel B shows the results of the fixed-effects model calculation, E = -0.3246, 95% confidence interval ranging from -0.3429 to -0.3063, Q (df = 25) = 1916.5405, $p < 0.0001$, $I^2 = 98.70\%$. Black squares represent the values of the cumulative effect size for each variable. Black horizontal lines represent the 95% confidence interval. Red solid lines indicate the cumulative effect values.)

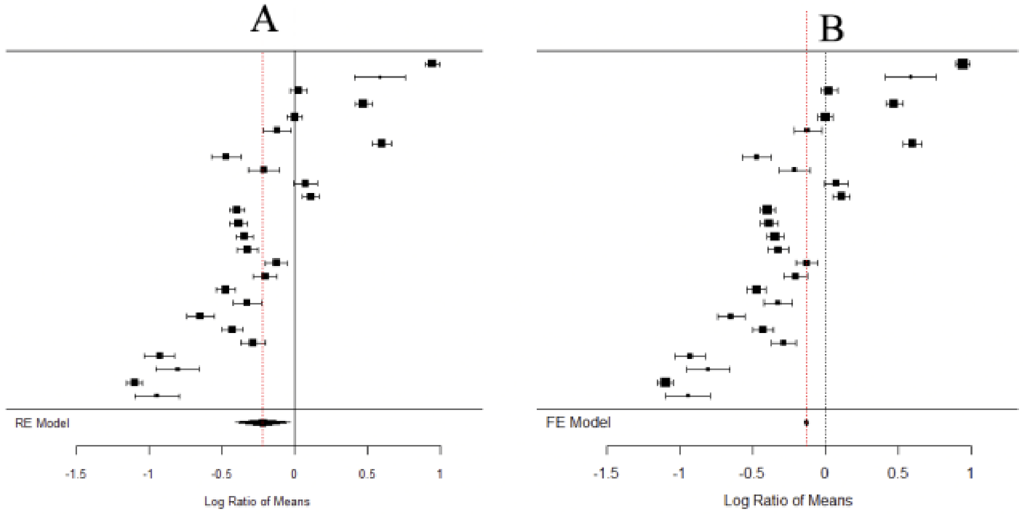

Fig. 3S Comparison of effect sizes between random-effects and fixed-effects models (Panel A displays the results of the random-effects model calculation, E = -0.2187, 95% confidence interval ranging from -0.4079 to -0.0296, Q (df = 25) = 5202.7682, $p <$ 0.0001, I^2 = 99.44%. Panel B shows the results of the fixed-effects model calculation, E = -0.1311, 95% confidence interval ranging from -0.1451 to -0.1170, Q (df = 25) = 5202.7682, $p < 0.0001$, I^2 = 99.52%. Black squares represent the values of the cumulative effect size for each variable. Black horizontal lines represent the 95% confidence interval. Red solid lines indicate the cumulative effect values.)

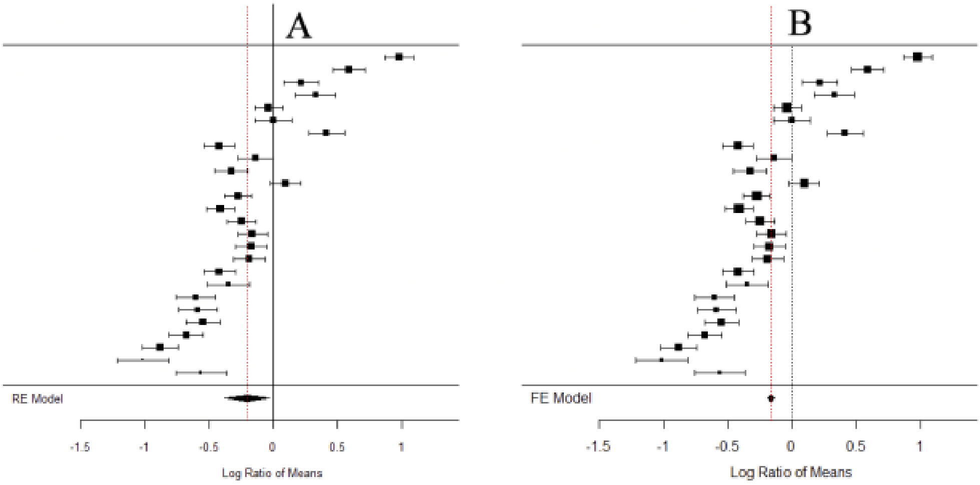

Fig. S4 Comparison of effect sizes between random-effects and fixed-effects models (Panel A displays the results of the random-effects model calculation, E = -0.2058, 95% confidence interval ranging from -0.3792 to -0.0324, Q (df = 25) = 1119.8463, $p <$ 0.0001, $I^2 = 97.85\%$. Panel B shows the results of the fixed-effects model calculation, E = -0.1633, 95% confidence interval ranging from -0.1187 to -0.1380, Q (df = 25) = 1119.8463, $p < 0.0001$, $I^2 = 97.77\%$. Black squares represent the values of the cumulative effect size for each variable. Black horizontal lines represent the 95% confidence interval. Red solid lines indicate the cumulative effect values.)

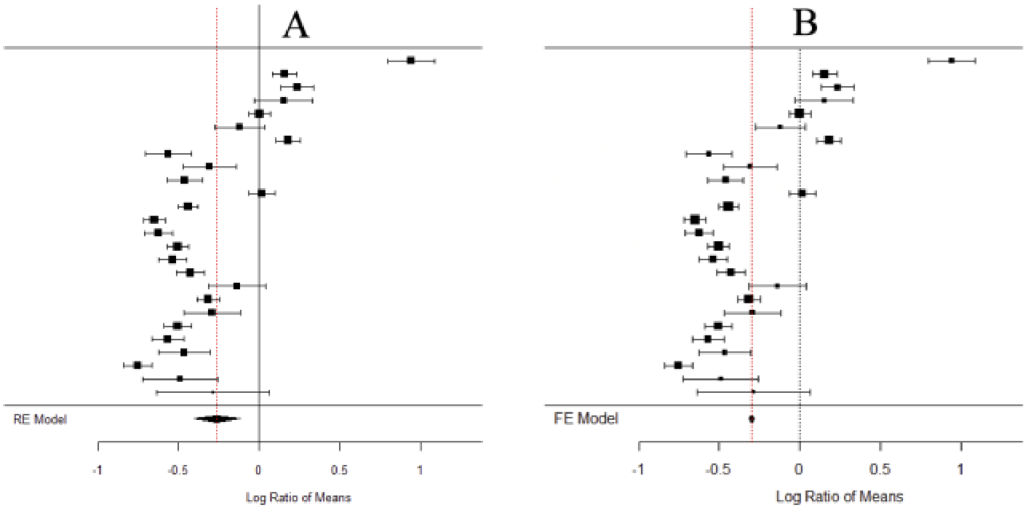

Fig. S5 Comparison of effect sizes between random-effects and fixed-effects models (Panel A displays the results of the random-effects model calculation, E = -0.2614, 95% confidence interval ranging from -0.4065 to -0.1163, Q (df = 25) = 1274.5347, p < 0.0001, I^2 = 98.29%. Panel B shows the results of the fixed-effects model calculation, E = -0.2990, 95% confidence interval ranging from -0.3176 to -0.2803, Q (df = 25) = 1274.5347, p < 0.0001, I^2 = 98.04%. Black squares represent the values of the cumulative effect size for each variable. Black horizontal lines represent the 95% confidence interval. Red solid lines indicate the cumulative effect values.)

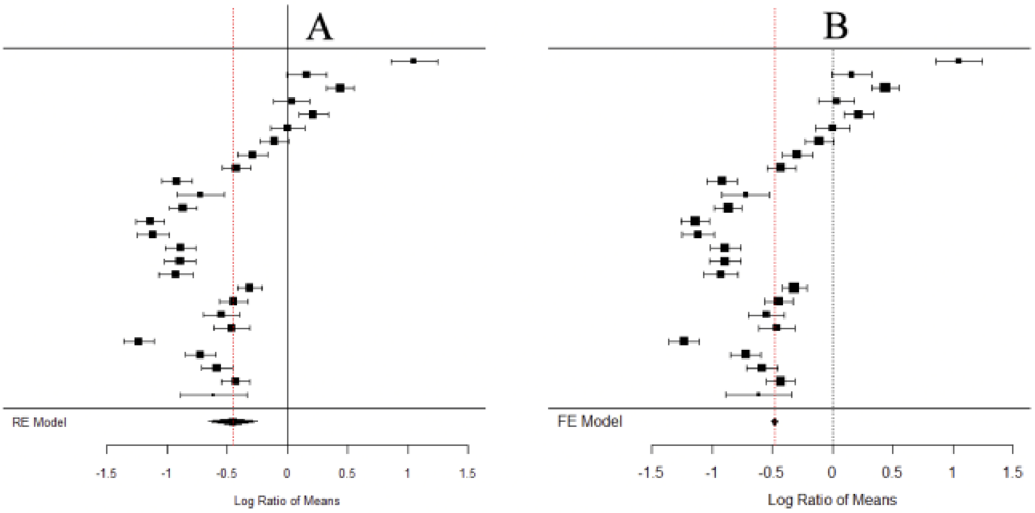

Fig. S6 Comparison of effect sizes between random-effects and fixed-effects models (Panel A displays the results of the random-effects model calculation, E = -0.4531, 95% confidence interval ranging from -0.6587 to -0.2475, Q (df = 25) = 1420.3349, $p <$ 0.0001, I^2 = 98.42%. Panel B shows the results of the fixed-effects model calculation, E = -0.4810, 95% confidence interval ranging from -0.5067 to -0.4553, Q (df = 25) = 1420.3349, $p < 0.0001$, $I^2 = 98.24\%$. Black squares represent the values of the cumulative effect size for each variable. Black horizontal lines represent the 95% confidence interval. Red solid lines indicate the cumulative effect values.)

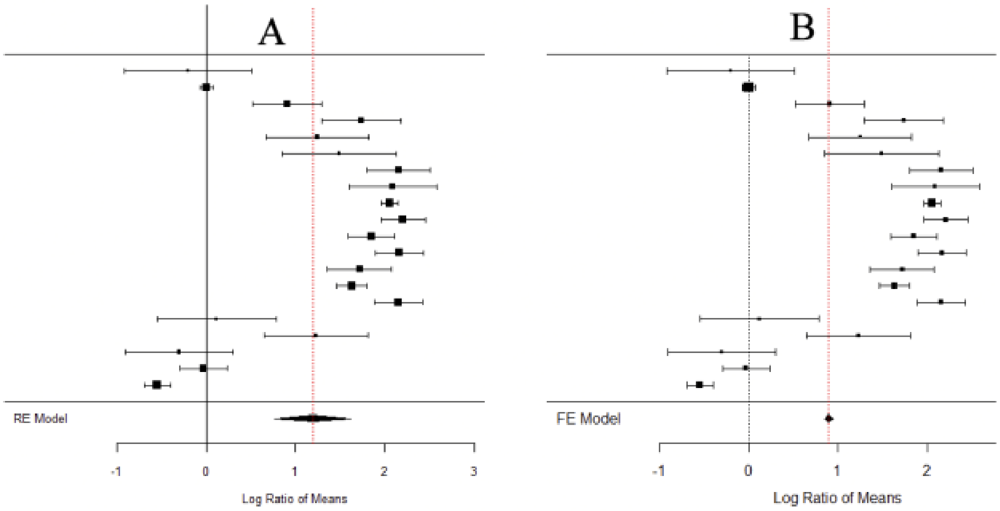

Fig. S7 Comparison of effect sizes between random-effects and fixed-effects models (Panel A displays the results of the random-effects model calculation, E = 1.1927, 95% confidence interval ranging from 0.7645 to 1.6209, Q (df = 19) = 2035.8560, p < 0.0001, I^2 = 99.07%. Panel B shows the results of the fixed-effects model calculation, E = -0.3246, 95% confidence interval ranging from -0.8472 to -0.9383, Q (df = 19) = 2035.8560, p < 0.0001, I^2 = 99.07%. Black squares represent the values of the cumulative effect size for each variable. Black horizontal lines represent the 95% confidence interval. Red solid lines indicate the cumulative effect values.)

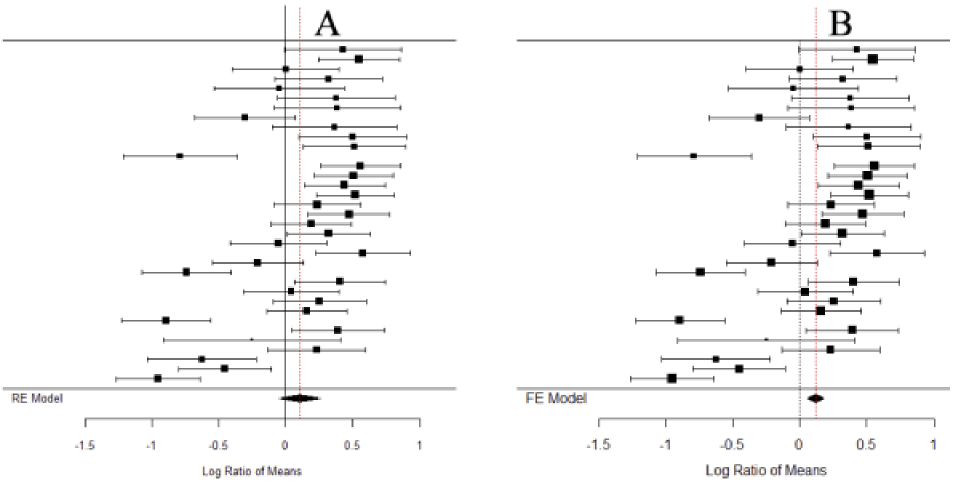

Fig. S8 Comparison of effect sizes between random-effects and fixed-effects models (Panel A displays the results of the random-effects model calculation, E = 0.1019, 95% confidence interval ranging from -0.0512 to -0.2551, Q (df = 34) = 226.5584, $p <$ 0.0001, $I^2 = 84.55\%$. Panel B shows the results of the fixed-effects model calculation, E = 0.1203, 95% confidence interval ranging from 0.0606 to 0.1799, Q (df = 34) = 226.5584, $p < 0.0001$, $I^2 = 84.99\%$. Black squares represent the values of the cumulative effect size for each variable. Black horizontal lines represent the 95% confidence interval. Red solid lines indicate the cumulative effect values.)

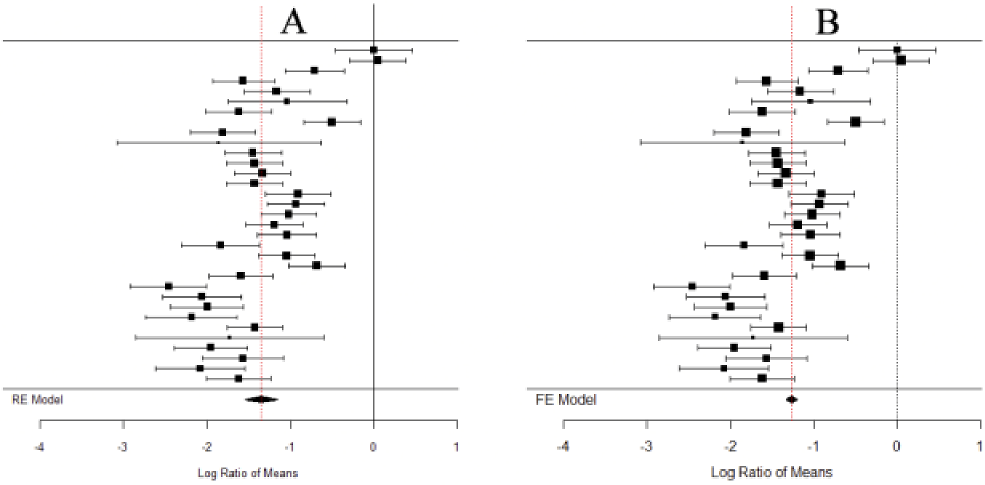

Fig. S9 Comparison of effect sizes between random-effects and fixed-effects models (Panel A displays the results of the random-effects model calculation, E = -1.3465, 95% confidence interval ranging from -1.5491 to -1.1438, Q (df = 32) = 250.5705, $p <$ 0.0001, I^2 = 87.96%. Panel B shows the results of the fixed-effects model calculation, E = -1.2656, 95% confidence interval ranging from -1.3345 to -1.1967, Q (df = 32) = 250.5705, $p < 0.0001$, I^2 = 87.23%. Black squares represent the values of the cumulative effect size for each variable. Black horizontal lines represent the 95% confidence interval. Red solid lines indicate the cumulative effect values.)

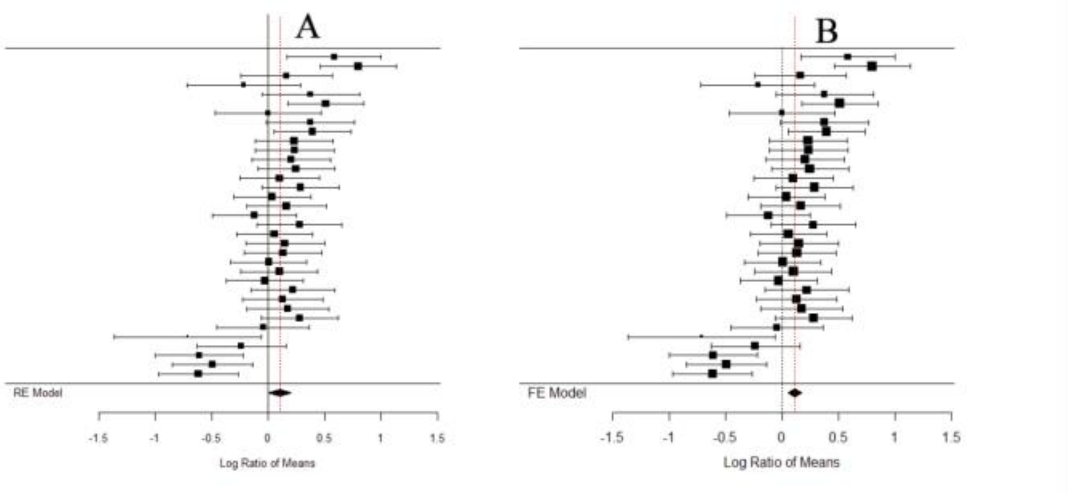

Fig. S10 Comparison of effect sizes between random-effects and fixed-effects models (Panel A displays the results of the random-effects model calculation, E = 0.1040, 95% confidence interval ranging from 0.0010 to 0.2070, Q (df = 34) = 92.4024, $p < 0.0001$, $I^2 = 63.28\%$. Panel B shows the results of the fixed-effects model calculation, E = 0.1151, 95% confidence interval ranging from 0.0531 to 0.1770, Q (df = 34) = 92.4024, $p < 0.0001$, $I^2 = 63.20\%$. Black squares represent the values of the cumulative effect size for each variable. Black horizontal lines represent the 95% confidence interval. Red solid lines indicate the cumulative effect values.)

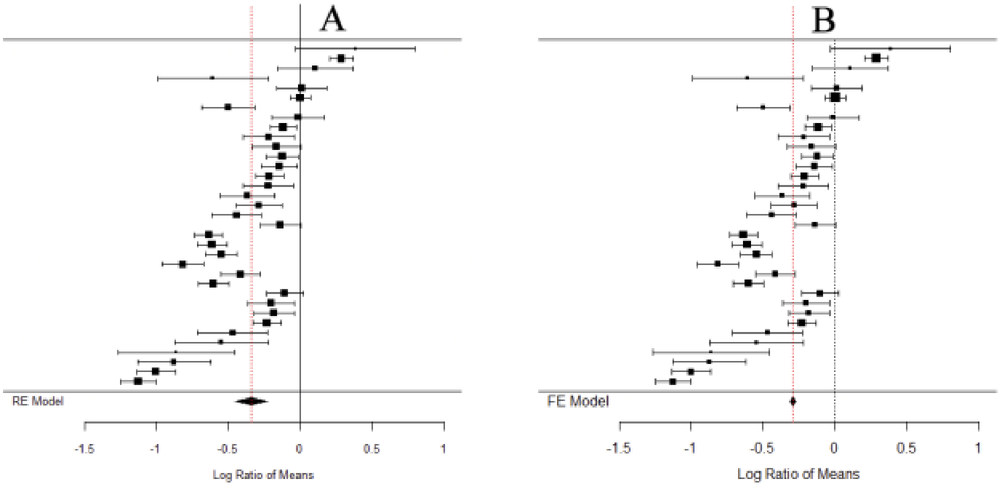

Fig. S11 Comparison of effect sizes between random-effects and fixed-effects models (Panel A displays the results of the random-effects model calculation, E = -0.3409, 95% confidence interval ranging from -0.4535 to -0.2283, *Q* (df = 34) = 881.2395, $p <$ 0.0001, $I^2 = 95.82\%$. Panel B shows the results of the fixed-effects model calculation, E = -0.2924, 95% confidence interval ranging from -0.3148 to -0.2700, *Q* (df = 34) = 881.2395, $p < 0.0001$, $I^2 = 96.14\%$. Black squares represent the values of the cumulative effect size for each variable. Black horizontal lines represent the 95% confidence interval. Red solid lines indicate the cumulative effect values.)

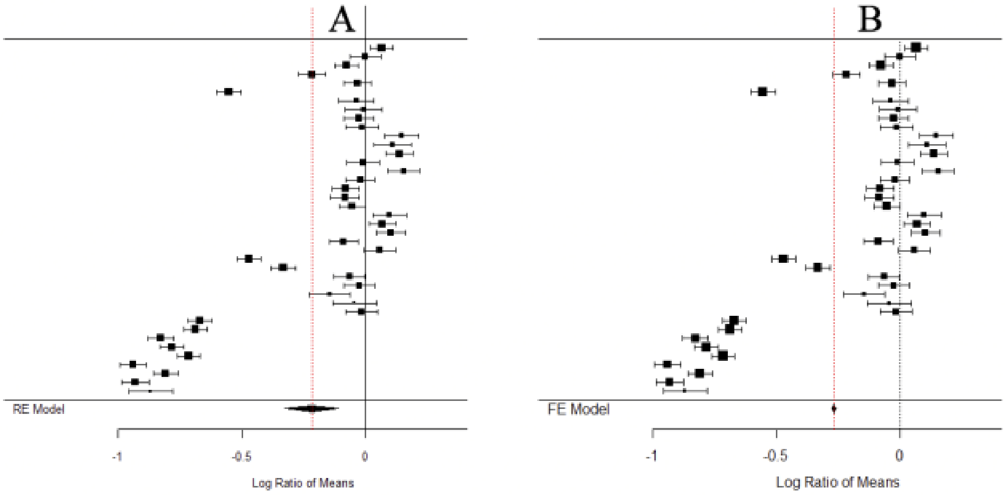

Fig. S12 Comparison of effect sizes between random-effects and fixed-effects models (Panel A displays the results of the random-effects model calculation, E = -0.0736, 95% confidence interval ranging from -0.1529 to 0.0056, Q (df = 108) = 3395.4298, p < 0.0001, I^2 = 96.51%. Panel B shows the results of the fixed-effects model calculation, E = -0.1144, 95% confidence interval ranging from -0.1291 to -0.0996, Q (df = 108) = 3395.4298, p < 0.0001, I^2 = 96.82%. Black squares represent the values of the cumulative effect size for each variable. Black horizontal lines represent the 95% confidence interval. Red solid lines indicate the cumulative effect values.)

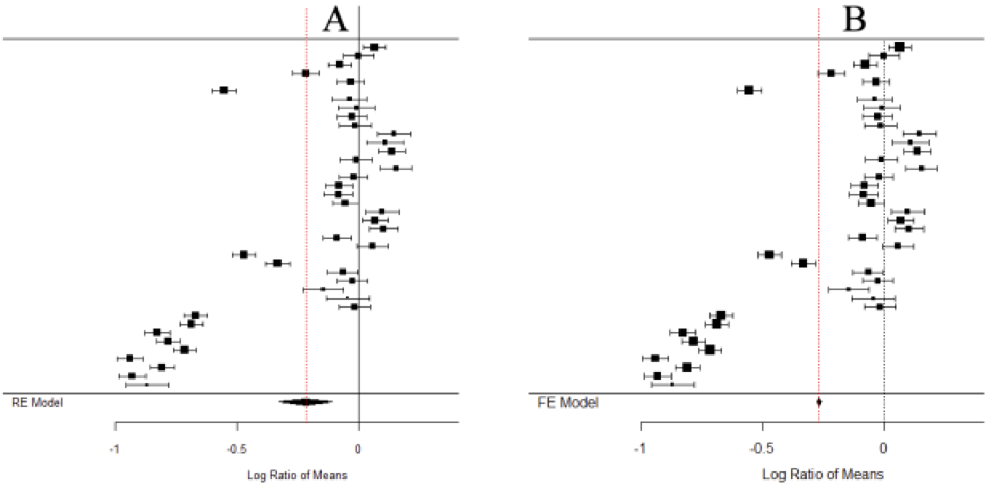

Fig. S13 Comparison of effect sizes between random-effects and fixed-effects models (Panel A displays the results of the random-effects model calculation, E = -0.2171, 95% confidence interval ranging from -0.3265to -0.1077, Q (df = 39) = 6001.3758, p < 0.0001, I^2 = 99.31%. Panel B shows the results of the fixed-effects model calculation, E = -0.2667, 95% confidence interval ranging from -0.2758 to -0.2576, Q (df = 39) = 6001.3758, p < 0.0001, I^2 = 99.35%. Black squares represent the values of the cumulative effect size for each variable. Black horizontal lines represent the 95% confidence interval. Red solid lines indicate the cumulative effect values.)

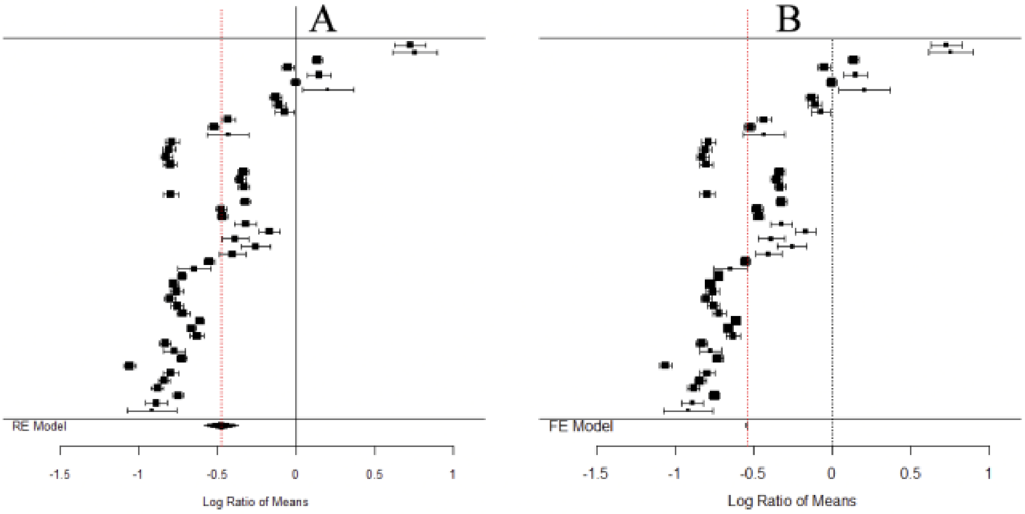

Fig. S14 Comparison of effect sizes between random-effects and fixed-effects models (Panel A displays the results of the random-effects model calculation, E = -0.4748, 95% confidence interval ranging from -0.5849 to -0.3648, Q (df = 49) = 10318.0476, $p <$ 0.0001, $I^2 = 99.71\%$. Panel B shows the results of the fixed-effects model calculation, E = -0.5443, 95% confidence interval ranging from -0.5502 to -0.5385, Q (df = 49) = 10318.0476, $p < 0.0001$, $I^2 = 99.53\%$. Black squares represent the values of the cumulative effect size for each variable. Black horizontal lines represent the 95% confidence interval. Red solid lines indicate the cumulative effect values.)
